# Practices of Procedural Pain Management in Neonates through Continuous Quality Improvement Measures

**DOI:** 10.1155/2022/8605071

**Published:** 2022-02-13

**Authors:** Jigar P. Thacker, Deep S. Shah, Dipen V. Patel, Somashekhar M. Nimbalkar

**Affiliations:** ^1^Department of Pediatrics, Pramukhswami Medical College, Bhaikaka University, Karamsad, Dist. Anand, Gujarat, India; ^2^Department of Neonatology, Pramukhswami Medical College, Bhaikaka University, Karamsad, Dist. Anand, Gujarat, India

## Abstract

**Objective:**

Although the benefits of pain control measures in neonates are well known, the actual usage was not optimal in our unit. Therefore, we implemented a quality improvement project to improve pain management practices through multiple Plan-Do-Study-Act (PDSA) cycles.

**Method:**

Our project included hemodynamically stable newborns weighing ≥1300 g. We identified four common procedures: intravenous cannulation, venous sampling, heel prick, and nasogastric tube insertion. The selected pain control measures were skin-to-skin contact, breastfeeding, expressed breast milk orally, and oral sucrose. Between April 2019 and September 2019, we intervened multiple times and reassessed shortcomings. We encouraged evidence-based practices and gave solutions for shortcomings. Data were interpreted weekly to assess the compliance to pain control interventions.

**Results:**

Minimal pain control measures (3-4%) were utilized for identified procedures before the project began. We could improve the use of pain control measures steadily and achieve the target of 80% of procedures after seven different interventions over five months. There was a retention of the effect on reassessing twice at second and fourth months of stopping further intervention once the target got achieved.

**Conclusion:**

Quality Improvement science can identify the shortcomings and help to improve the compliance for pain control practices in neonates, as demonstrated in this neonatal unit.

## 1. Introduction

The neuroanatomical and neurobiochemical maturation of neonates to perceive painful and nociceptive stimuli were demonstrated by the mid-1980s [1, 2]. Irrespective of gestational age, painful stimuli in neonates are reflected in cardiorespiratory, behavioral, hormonal, and metabolic changes to a similar extent or more intensely than in children or adults [1, 3, 4]. The long-term deleterious effects of neonatal pain are altered neurodevelopment of the pain perceiving system and disturbed social, emotional, and psychological functioning [1, 5]. Though measuring and quantifying painful experiences in neonates objectively is difficult, various standardized pain scales have been studied. Most of them consider vital signs, breathing patterns, facial expressions, and crying [6–9].

Neonates undergo multiple painful experiences during their stay in the nursery [10]. Neonatal pain should be prevented whenever possible, and pain control interventions must be implemented when it is unavoidable. Therefore, it is necessary to provide pain limiting measures while performing invasive procedures. Many nonpharmacological and pharmacological pain control interventions have been adopted over time. The nonpharmacological measures are as follows: breastfeeding or expressed breast milk (EBM), skin to skin contact (SSC) or kangaroo mother care (KMC), swaddling, and nonnutritive sucking, among others. The pharmacological measures include oral sucrose (24% solution), local or topical anesthesia, and systemic drugs like acetaminophen, nonsteroidal anti-inflammatory drugs (NSAIDs), opioids, or general anesthetics [11–13]. It was observed that positional measures like swaddling, facilitated tucking, or nonnutritive sucking are less effective on their own but synergize the effect of sweetened solutions like sucrose [12]. When a procedure is performed, analgesia can be provided in a tiered manner with the increasing requirement from tier 1 consisting of nonpharmacological measures to tier 5, which includes deep sedation or anesthesia [12]. Standard guidelines have been proposed for procedural pain management in neonates [13, 14]. Although our unit had conducted several studies addressing neonatal pain management [15–17], the actual practice remained suboptimal, particularly in the level II nursery. In a review, Cruz et al. found that pain management was inadequately utilized for the frequently performed procedures in neonates. The organizational factors may be modified for favorable pain management practice [18]. Similar clinical issues have been reported worldwide with the call for a systematic approach for neonatal procedural pain management [10, 19, 20]. Adult care also acknowledges that a deficit persists in pain management practices. Such deficits are not due to the lack of medical knowledge or complexity of a medical condition but rather due to the complexity of hospital structure and workflow organization. To overcome this lacuna, continuous quality improvement measures have been adopted [21, 22].

Quality improvement (QI) principles have improved neonatal pain management practices [23, 24]. In addition, the World Health Organization's (WHO) 2007 Framework for Action for strengthening health systems in developing countries identified quality as one of the key drivers for improved health outcomes and greater efficiency in health service delivery [25]. Therefore, we utilized the QI framework to improve pain control interventions while performing common neonatal procedures.

## 2. Methods

### 2.1. Context

The project was conducted in a university teaching hospital having 26 bedded neonatal intensive care unit (NICU) providing level II and III care, with around 550 admissions per year with 70% bed occupancy. Three neonatal physicians, two fellows, seven residents, and 26 neonatal nurses provide clinical care. The nurse-patient ratio is 1 : 3 for stable newborns and 1 : 1 for critical newborns, and we reduce patient inflow if enough nurses are not available due to any exigencies. The team observed that pain control measures were overlooked in a comparatively stable newborn undergoing various routine invasive procedures.

### 2.2. Interventions

Hemodynamically stable newborns weighing more than 1300 g, without respiratory distress or shock, who were neurologically intact were included in the study. Newborns with any surgical condition were excluded. Before implementing the project, in March 2019, our QI team conducted a cross-sectional assessment of around 120 health records of 20 patients and identified four commonly performed invasive procedures: heel prick for random blood sugar (RBS) monitoring, intravenous (IV) sampling, IV cannulation, and nasogastric (NG) tube insertion. During the process assessment, pain control measures were often underutilized in the given subset of patients undergoing selected procedures. After reviewing the literature [12, 14] and assessing the local feasibility, we identified four feasible pain control interventions while performing identified invasive procedures: skin to skin contact or KMC, direct breastfeeding, EBM by mouth, and oral sucrose. We formed a quality improvement team to address this issue: three physicians, one neonatal fellow, three residents, and five nurses.

### 2.3. Study of Interventions

Multiple small Plan-Do-Study-Act (PDSA) cycles are the basis for the QI project. Once the problem is prioritized, the system is analyzed at multiple levels, and a potential intervention is planned and implemented. The outcome is then tested for change. These interventions continue until the feasible goal is achieved [26, 27].

### 2.4. Measures

Our key outcome measure was the proportion of occasions in any of the pain control interventions taken prior to performing any of the four identified invasive procedures. Data were collected for each such occasion and expressed weekly in a percentage proportion. One nurse and one resident or fellow were given the responsibility of data collection during all three shifts. Neonates were identified on the morning of each day based on inclusion criteria. The baseline data collection was conducted for two weeks starting from 15^th^ April 2019. Then, data collection was continued throughout the study period, and three more weeks after, we achieved the target of 80% for the first time during the week of 16^th^ September to 22^nd^ September 2019. Finally, retention analysis was done twice for two weeks period in the last half of December 2019 and the first half of February 2020.

Every attempt was made to minimize the missing data. As mentioned, nursing staff and a resident were given the task of data collection individually. Any discrepancy in their data was sorted out with one on one inquiry by a physician on the next day. The unit's nursing policy was to document each RBS in the nursing chart. The IV sampling, IV cannula, or NG tube insertion are not frequent in included stable subsets of patients, and each such occasion can easily be notified. Still, those occasions were cross verified with the pharmacy purchase of NG tube or IV cannula and laboratory test entry whenever necessary.

### 2.5. Analysis

On analyzing the system, the first problem identified was the health care team's lack of awareness and education about neonatal pain management. Thus, in the beginning, the first intervention was an educational session in the form of two evidence-based lectures lasting 45 minutes each, conducted twice on subsequent days. The content of the lectures included but was not limited to the physiological basis of neonatal pain, serious clinical consequences of untreated pain and thus the need to tackle it, the fact that it is often being overlooked, and how the selected pain control measures can be implemented while performing the identified four procedures. In addition, short videos of newborns' facial expressions while performing painful procedures were shown to sensitize the staff about pain perception. Study authors conducted the session in a comfortable, friendly setting where the participants could share their ideas. On reassessing, it is seen that nurses tend to forget to practice pain control measures in their routine work patterns. Two approaches were implemented at close intervals: (a) a column was added in a monitoring chart for nurses to mention pain control intervention for the particular procedure. Nurses wrote a painful procedure and the pain control intervention given in this column based on the feedback given by investigators. (b) A poster reminder showing four painful procedures and suitable options for pain control interventions was pinned in the nursing and patient care area as a guide while performing a particular procedure. The regular biweekly meetings were started to identify any system resource issues, boost motivation, and track progress in this regard. At this stage, it was observed that frequent sucrose utilization added extra cost to the parents. So, it was decided to use EBM as the first choice to balance this cost issue whenever direct breastfeeding was not feasible.

**Figure 1:**
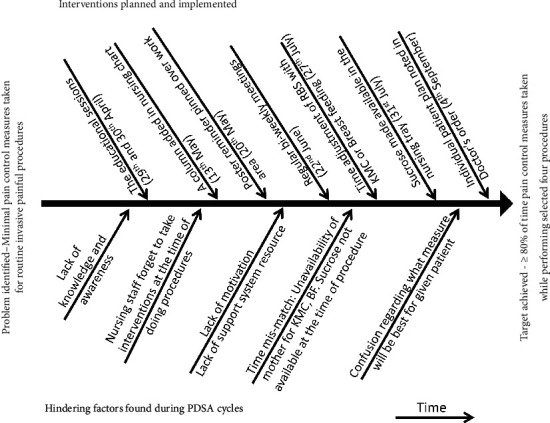
Diagram showing the interventions for hindering factors over time.

There was a problem of time mismatch. While performing the required invasive procedure, the mother was unavailable for KMC or breastfeeding, or the sucrose vial was unavailable. To address this, the routine RBS was adjusted with the closest nearby time of the KMC session or breastfeeding time so that ongoing care was not hampered. Furthermore, a buffer stock of oral sucrose was made available in a central location for supplies (nursing tray). Lastly, we made an individualized patient plan whenever necessary and feasible to overcome confusion about what measure would be best suited while doing a given invasive procedure. Likewise, we write in a doctor's order, “Do RBS twice a day with 1 ml EBM orally 2 minutes prior.” The target of 80% was achieved for the first time in the week of 16^th^ September to 22^nd^ September 2019.  Figure 1 showcases problems identified in the system towards the achievement of the goal and interventions made to overcome those problems. Following this, no further intervention was planned, and retention analysis was done.

### 2.6. Ethical Considerations

The project was submitted and approved by the Institutional Ethics Committee. It was conveyed clearly at the outset of the process and frequently reiterated that this project was to improve pain control practices in the department as a whole, and no constructive criticism was intended personally. We ensured transparency in data collection and communication among all levels of health care staff, and the study team constantly supervised it. During this project, flaws identified had not affected the staff's departmental or institutional appraisal, nor had their strengths been rewarded.

## 3. Results

**Figure 2:**
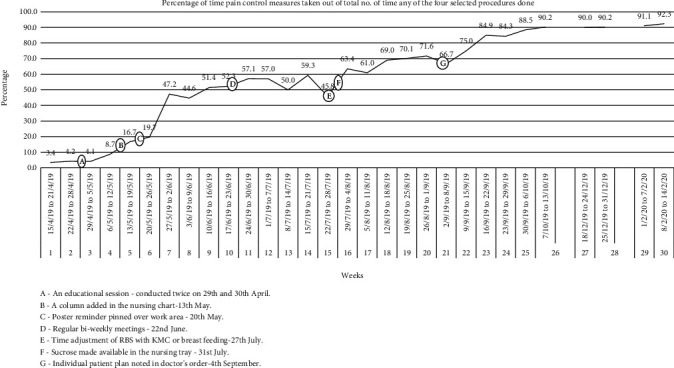
Percentage of time pain control measures taken out of total no. of time in any of the four selected procedures done.

Table 1 shows the weekly data with the actual numbers of times each of the selected painful procedures performed, with the actual numbers of times in any of the selected pain control measures used with the proportion.  Figure 2 shows the timeline diagram of compliance. At baseline, the proportion of pain control measures utilized during the four invasive procedures was negligible at 3-4%. There was some change after the first educational intervention, and the compliance improved up to 16.7% over the next two weeks. The 2^nd^ (column added in the monitoring chart for nurses) and 3^rd^ (poster reminder pinned at nursing work area) interventions significantly improved up to 47.2% instantaneously. These interventions provided a real-time reminder at the actual time and place of performing the procedure. It seems that the 4^th^ intervention of regular biweekly meetings did not lead to significant gain but that helped us better understand the system, and we came to know about the problem of time mismatch. Overcoming the problems of time mismatch and unavailability of sucrose in 5^th^ (time adjustment of the procedure with that of KMC or breastfeeding) and 6^th^ (sucrose made available in a central supply location) interventions led to further steady improvement.

Within the two weeks of making an individualized patient plan as a 7^th^ intervention on 4^th^ September 2019, our target of 80% was achieved for the first time in the week of 16^th^ to 22^nd^ September 2019. We discontinued the biweekly meetings at this time. The column added in the nurses' monitoring chart or poster reminder pinned remained as it is. We continued the improved practice of time adjustment, sucrose availability, and making an individualized patient pain control plan. We did not find any specific health provider characteristic affecting the compliance.

On subgroup analysis of the data, it became evident that even though the overall target of 80% had been achieved, the practice was not established for the NG tube insertion. On root cause analysis, we found that nurses believed that anything should not be given orally when an NG tube was being inserted. It was clarified on 30^th^ September that giving just 1 ml of EBM or sucrose 2 minutes before NG tube insertion cannot be considered as a feed, and it is similar to trophic feeds. After this explanation, pain management measures in NG tube insertion escalated. The effect was then retained and did not deescalate on subsequent data collections.

A total of 2036 selected painful procedures were performed during the entire observational period. Out of these, the selected pain control interventions were done for 1096 occasions, and in the decreasing order of the frequency, they were EMB—532, sucrose—340, direct breastfeeding—219, and KMC—5.

## 4. Discussion

We successfully implemented a quality improvement project to improve procedural pain management in neonates in our neonatal unit. We attempted to bridge the knowledge-practice gap by systemically identifying the obstacles causing shortfalls from our target, then planning solutions, and implementing them. It was apparent that no single intervention could lead to significant and consistent results, but it was only possible with further interventions implemented, and the earlier one was reinforced. Slowly, the working practices within the unit changed, and the desired practice was established as a routine.

Significant research studies in the past were foundational for neonatal pain assessment and management. The International Evidence-Based Group for Neonatal Pain established the guidelines for neonatal pain management in a consensus statement twenty years ago [11]. A pain study group of the Italian Society of Neonatology gave guidelines in a review article for procedural pain in newborns [13]. American Academy of Pediatrics updated the prevention and management of procedural pain in neonates as a policy statement [14]. A guide to pain assessment and a tiered approach for pain management were suggested [12].

Despite these efforts, there are limited studies that support how to implement these guidelines into practice. A large cross-sectional study, the Epidemiology of Procedural Pain in Neonates (EPIPPAIN) conducted in 2005-06 in France, involving 430 neonates with 42413 painful procedures, identified that neonatal analgesia was not provided for a majority of the occasions [10]. Years later, the EPIPPAIN 2 project conducted in 2011 identified that it was not systematic or uniform for the commonest procedures like heel sticks and venipuncture, even though analgesic use was improved. Still, there was a scope for improvement [28, 29]. A recent systematic review again reported infrequent utilization of the neonatal pain management strategies [18]. Thus, the gap between research findings and clinical practice exists, and there is a need to develop methods to facilitate the effective implementation of neonatal analgesia. Some studies showed an improved perception and understanding of health care staff over neonatal pain and its management after an educational intervention [15, 30]. However, an improved understanding does not always reflect in practice. In another study, significant improvement in sucrose order documentation and administration was observed with implementing a process evaluation checklist [31]. In Australia, a nationwide Practice Evidence Gap Strategy (PEGS) project was launched in 2006 to improve pain management practices, continued resource educational support was provided, and the local champions were identified. A multisite survey on the completion of the project in 2012 noted improved awareness and use of sucrose and breastfeeding for procedural pain [32].

Two multicentric trials applied QI principles to improve neonatal pain management in the involved sites [23, 24]. In both the studies, local groups were created, better practices were identified and implemented, and improvement over time at different involved sites was assessed but did not discuss site-specific processes or obstacles faced to implement the practices, as the current study does. We set an example of how an individual neonatal unit can adopt a quality improvement process for procedural neonatal pain management. There is evidence of benefit with continuous quality improvement efforts for pain management in the intensive care unit (ICU) and postoperative settings. Pasero et al. noted that “A multidisciplinary and patient-centered continuous quality improvement process is essential to identifying barriers and implementing evidence-based solutions to the problem of undertreated pain in hospital ICUs” [33]. Another study in postoperative setup successfully used a continuous quality improvement method with frequent assessments of process and outcome parameters, regular benchmarking, and feedback mechanisms. They concluded that changes in organization and multidisciplinary teamwork are more important than medical or technical aspects [21].

We identified pain control measures based on the earlier guideline for procedural pain management and as per our local feasibility. The recent meta-analysis and systematic review published in 2020 by Wade et al. recommended breastfeeding as a first-line intervention. They also added that whenever breastfeeding is not possible, 1–2 mL of EMB should be used as first-line followed by 1–2 mL of oral sugar as a second-line analgesic [34]. Contrary to this observation, the earlier review by Benoit et al. questioned the effectiveness of EBM alone as an effective pain control intervention [35]. Shah et al., in their Cochrane review, observed that EBM was more effective than no intervention or placebo but was inferior compared to direct breastfeeding and sweet solutions. If direct breastfeeding is not feasible, the most effective alternative strategy with a similar effect to reduce pain during acute painful procedures is oral glucose/sucrose solution [36]. Both of these studies could not conclude the amount of EBM needed to relieve pain. In our study, we used a small amount (1-2 ml) of EBM, which might be less effective than the larger volume. This is further needed to be studied.

KMC and breastfeeding have been promoted and practiced in our unit for more than two decades. Thus, it was feasible for us to adjust the time of heel prick with KMC and breastfeeding. Sucrose was also available in our pharmacy, but we ensured its presence in the nursing tray at the bedside for ready use, and then, it was replaced subsequently. We introduced pain management advice as a direct physician order in the last step. As per the survey, the nurses perceived that insufficient physician order was the lead cause for the undertreatment of pediatric pain [37].

Our project is an example to implement the QI method to address one of the problems found in the actual practice. We hope to implement principles of QI in other neonatal care areas. We could sustain the practice on retention analysis. Subsequent periodic evaluations should be continued to withstand the practices.

### 4.1. Limitations

This project design may not be generalizable to all neonatal units. With the different baseline practices, patterns of staff, and infrastructure availability, each unit can modify and implement its steps. We could not monitor the impact of improved practice in terms of improvement in pain score or vital stability of the newborns clinically. We did not conduct interviews of parents or health care professionals involved in the project. Due to feasibility issues, we used EBM as a preferred intervention to KMC or oral sucrose when direct breastfeeding was not feasible.

## 5. Conclusion

Knowledge about evidence-based neonatal pain management recommendations may not be reflected in neonatal care. A quality improvement model may be helpful to bridge the knowledge-practice gap, as the current project demonstrated in our NICU for procedural pain management.

## Figures and Tables

**Table 1:** Weekly data with subgroup analysis.

SR. no.	Weeks	RBS			IV cannulation			IV sample			NG tube insertion				Total	
		A	B	%	A	B	%	A	B	%	A	B	%	A	B	%
1	15/4/19 to 21/4/19	87	4	4.6	6	0	0.0	10	0	0.0	15	0	0.0	118	4	3.4
2	22/4/19 to 28/4/19	70	3	4.3	5	0	0.0	7	1	14.3	13	0	0.0	95	4	4.2
3	29/4/19 to 5/5/19	36	1	2.8	4	1	25.0	4	0	0.0	5	0	0.0	49	2	4.1
4	6/5/19 to 12/5/19	31	2	6.5	5	1	20.0	5	1	20.0	5	0	0.0	46	4	8.7
5	13/5/19 to 19/5/19	52	10	19.2	4	0	0.0	5	1	20.0	5	0	0.0	66	11	16.7
6	20/5/19 to 26/5/19	48	12	25.0	4	0	0.0	5	0	0.0	4	0	0.0	61	12	19.7
7	27/5/19 to 2/6/19	48	31	64.6	5	1	20.0	7	2	28.6	12	0	0.0	72	34	47.2
8	3/6/19 to 9/6/19	56	31	55.4	4	0	0.0	4	2	50.0	10	0	0.0	74	33	44.6
9	10/6/19 to 16/6/19	51	34	66.7	5	0	0.0	5	2	40.0	9	0	0.0	70	36	51.4
10	17/6/19 to 23/6/19	50	34	68.0	4	0	0.0	4	0	0.0	7	0	0.0	65	34	52.3
11	24/6/19 to 30/6/19	94	63	67.0	4	1	25.0	8	4	50.0	13	0	0.0	119	68	57.1
12	1/7/19 to 7/7/19	87	56	64.4	3	1	33.3	8	4	50.0	9	0	0.0	107	61	57.0
13	8/7/19 to 14/7/19	57	35	61.4	4	1	25.0	3	0	0.0	8	0	0.0	72	36	50.0
14	15/7/19 to 21/7/19	70	49	70.0	4	1	25.0	3	1	33.3	9	0	0.0	86	51	59.3
15	22/7/19 to 28/7/19	54	28	51.9	5	2	40.0	6	3	50.0	7	0	0.0	72	33	45.8
16	29/7/19 to 4/8/19	31	21	67.7	4	2	50.0	5	3	60.0	1	0	0.0	41	26	63.4
17	5/8/19 to 11/8/19	61	41	67.2	6	4	66.7	7	5	71.4	8	0	0.0	82	50	61.0
18	12/8/19 to 18/8/19	47	38	80.9	5	3	60.0	10	8	80.0	9	0	0.0	71	49	69.0
19	19/8/19 to 25/8/19	53	41	77.4	3	2	66.7	6	4	66.7	5	0	0.0	67	47	70.1
20	26/8/19 to 1/9/19	52	41	78.8	5	3	60.0	5	4	80.0	5	0	0.0	67	48	71.6
21	2/9/19 to 8/9/19	42	31	73.8	4	4	100.0	5	5	100.0	9	0	0.0	60	40	66.7
22	9/9/19 to 15/9/19	44	38	86.4	4	3	75.0	5	4	80.0	7	0	0.0	60	45	75.0
23	16/9/19 to 22/9/19	67	62	92.5	5	5	100.0	6	6	100.0	8	0	0.0	86	73	84.9
24	23/9/19 to 29/9/19	38	36	94.7	5	4	80.0	4	3	75.0	4	0	0.0	51	43	84.3
25	30/9/19 to 6/10/19	39	37	94.9	6	5	83.3	4	3	75.0	3	1	33.3	52	46	88.5
26	7/10/19 to 13/10/19	36	35	97.2	4	4	100.0	6	5	83.3	5	2	40.0	51	46	90.2
27	18/12/19 to 24/12/19	28	27	96.4	3	3	100.0	5	4	80.0	4	2	50.0	40	36	90.0
28	25/12/19 to 31/12/19	29	28	96.6	3	3	100.0	5	4	80.0	4	2	50.0	41	37	90.2
29	1/2/20 to 7/2/20	40	38	95.0	4	4	100.0	4	4	100.0	8	5	62.5	56	51	91.1
30	8/2/20 to 14/2/20	30	29	96.7	2	2	100.0	3	3	100.0	4	2	50.0	39	36	92.3

Total number of each selected painful procedures done (A) and number of times in any of the pain control intervention taken (B) in an included subset of the neonates.

## Data Availability

The data are available with the corresponding author and will be provided on reasonable request.
